# Musashi-1 promotes a cancer stem cell lineage and chemoresistance in colorectal cancer cells

**DOI:** 10.1038/s41598-017-02057-9

**Published:** 2017-05-19

**Authors:** Guang-Yuh Chiou, Tzu-Wei Yang, Chi-Chou Huang, Chia-Ying Tang, Jung-Yi Yen, Ming-Chang Tsai, Hsuan-Yi Chen, Nurul Fadhilah, Chun-Che Lin, Yuh-Jyh Jong

**Affiliations:** 1 Department of Biological Science and Technology, College of Biological Science and Technology, National Chiao Tung University, Hsinchu, Taiwan; 2 Division of Gastroenterology and Hepatology, Department of Internal Medicine, Chung Shan Medical University Hospital, Taichung, Taiwan; 3 School of Medicine, Chung Shan Medical University, Taichung, Taiwan; 4 Division of Colon and Rectum, Department of Surgery, Chung Shan Medical University Hospital, Taichung, Taiwan; 5 Institute of Molecular Medicine and Bioengineering, College of Biological Science and Technology, National Chiao Tung University, Hsinchu, Taiwan; 6 Graduate Institute of Clinical Medicine, College of Medicine, Kaohsiung Medical University, Kaohsiung, Taiwan; 7 Departments of Paediatrics and Laboratory Medicine, Kaohsiung Medical University Hospital, Kaohsiung Medical University, Kaohsiung, Taiwan

## Abstract

Colorectal cancers (CRCs) are a critical health issue worldwide. Cancer stem cell (CSC) lineages are associated with tumour transformation, progression, and malignant transformation. However, how lineages are transformed and how chemoresistance is acquired by CRCs remain largely unknown. In this report, we demonstrated that the RNA-binding protein Musashi-1 enhanced the development of CD44^+^ colorectal CSCs and triggered the formation of anti-apoptotic stress granules (SGs). Our results indicated that CD44^+^ CSC lineage-specific induction of tumour malignancies was controlled by Musashi-1. In addition, Musashi-1 formed SGs when CRC cell lines were treated with 5-fluorouracil. The C-terminal domain of Musashi-1 was critical for recruitment of Musashi-1 into SGs. Intracellular Musashi-1 SGs enhanced the chemoresistance of CRCs. Analysis of clinical CRC samples indicated that Musashi-1 expression was prominent in CRC stage IIA and IIB. In summary, we demonstrated that *Musashi-1*, a stemness gene, is a critical modulator that promotes the development of CD44^+^ colorectal CSCs and also enhances CRC chemoresistance via formation of SGs. Our findings elucidated a novel mechanism of CRC chemoresistance through increased anti-apoptotic effects via Musashi-1-associated SGs.

## Introduction

Colorectal cancer (CRC) has a high incidence in developed countries. Morbidity and mortality from CRC are predominantly due to metastasis and drug resistance and are a critical threat to human health worldwide^1, 2^. Decades of research has shown that CRC transformations are regulated by multiple factors, including microsatellite instability, BRAF^V600E^, K-ras^G12D^, and p53^3^. Furthermore, aberrant crypt foci^4^ and mutated adenomatous polyposis coli^5^ are observed when CRC progresses into the adenoma stages. Increased expression of cyclooxygenase-2^6–8^, k-Ras^5, 9, 10^, and β-catenin has been observed in advanced clinical specimens^11, 12^; additionally, loss of p53^5, 13, 14^ and phosphatase and tensin homolog^15, 16^ and high levels of phosphatidylinositol 3-kinase have also been associated with CRC^17^.

Recently, Musashi-1 was identified as a novel regulator of CRC^18, 19^. Among stemness genes, *Musashi-1* is both a colon and neuronal stem cell marker. Musashi-1 contains two RNA recognition motifs (RRMs), RRM1 and RRM2, which bind to RNA molecules and act as translational repressors of, for example, p21^CIP^ and promote cellular proliferation^20, 21^.

Interestingly, environmental factors also contribute to CRC formation. Analyses of the molecular signatures of CRC development supported a two-hit hypothesis: loss of a tumour suppressor in the early stage and activation of oncogenes in the late stages^22^. Chronic inflammation triggers the production of reactive oxygen species, which, if prolonged, may activate pro-apoptotic pathways. Therefore, elucidating the mechanisms utilized by CRCs to escape from extracellular stress-induced cell death may increase the understanding of CRC malignancies and relapses.

Cancer relapses are associated with the development of drug resistance and acquisition of cancer stemness properties. Increasing evidence has shown that cancer cells are capable of escaping from cellular stresses. Stress granules (SGs)^23, 24^ are cytosolic ribonucleoprotein (RNP)-complexes that facilitate cellular stress resistance activities and are associated with specific diseases, including cancers. These processes are related to cellular vitalities under both stress and normal developmental conditions.

The ability of anti-apoptotic SGs to facilitate the escape of cancer cells from chemotherapy has been reported in many different cancer types. However, the association between SGs and tumourigenesis is unclear. Cancer stem cells (CSCs) are small cell populations that are capable of self-renewal and tumour-initiation properties within tumour tissues. CSCs are believed to be niches for refractory tumours, drug resistance, and malignancies^25^.

Various colorectal CSC surface markers have been identified, including CD133^26^, CD44^27–29^, and CD44v6, as well as the intracellular enzyme aldehyde dehydrogenase 1^30, 31^. In CRCs, a lineage-tracking technique in an animal model identified Lgr5 as an intestinal and colon stem cell surface marker^32^. Additionally, CRCs acquire stemness properties from environmental stimuli, such as IL-8^26^ and hypoxia^33^. Snail regulates IL-8 expression and facilitates the acquisition of stemness properties by colorectal cells^26^.

CD44, CD44v6, and Musashi-1 are considered to be CRC stem cell markers because their representative cellular populations overlap^34^. Furthermore, Musashi-1 maintains the CSC fate of CRC cells derived from xenografted tumours^34^. Direct evidence of Musashi-1-mediated regulation of CRCs came from knockdown experiments showing suppression of CRC progression^20^. Musashi-1 is located in the cytosol and participates in RNP complex formation. Therefore, it is important to determine whether Musashi-1 interacts with RNPs to regulate CRC progression.

In general, cancer cell plasticity can be induced by environmental factors, and cells adapt to environmental changes by transformation. Taken together, the available evidence supports the hypothesis that stress response factors may be linked to cancer cell plasticity and may provide answers to the problem of CRC drug resistance and transformation. The current study is designed to address this possibility.

## Results

**Figure 1:**
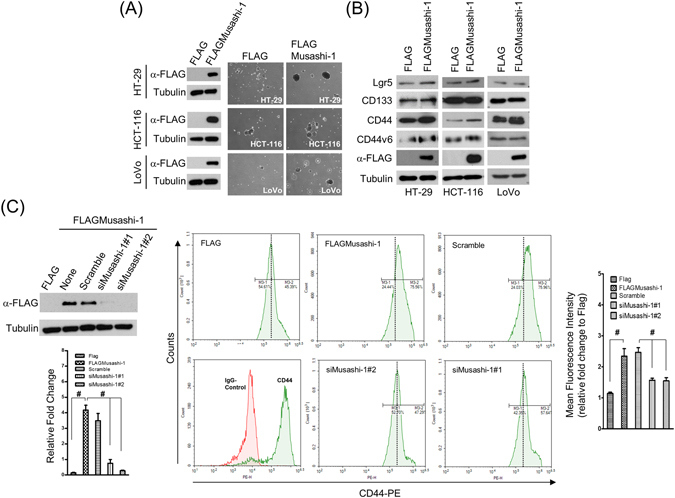
Musashi-1 promotes CD44^+^ CRC traits. (**A**) Establishment of Musashi-1-overexpressing CRC cells (FLAG/FLAGMusashi-1). HT-29, HCT-116, and LoVo cells were transfected with 3× FLAG and 3× FLAGMusashi-1 expression vectors, yielding the stable clones of HT-29, HCT-116, and LoVo cells with FLAG/FLAGMusashi-1, respectively. Stably transfected cells were selected by G418 (4 mg/mL) in culture medium for 4 weeks. Total protein of selected stable cell lines was obtained by lysis in RIPA buffer with protease and phosphatase inhibitors. Samples were subjected to immunoblotting analysis with a monoclonal anti-FLAG antibody (left panel). Spheroid formation was determined by culturing FLAG (HT-29, HCT-116, and LoVo cells) and FLAGMusashi-1 (HT-29, HCT-116, and LoVo cells) cells in spheroid formation buffer for 2 weeks. Images were acquired with Olympus cellSens software v1.12 (right panel). (**B**) Immunoblotting analysis of CSC marker expression in HT-29, HCT-116, and LoVo cells /FLAG and HT-29, HCT-116, and LoVo cells/FLAGMusashi-1 cells. Total cell lysates of FLAG- or FLAGMusashi-1-overexpressing stable cell lines were collected in RIPA lysis buffer. Total proteins were subjected to immunoblotting analyses with Lgr5-, CD133-, CD44-, and CD44v6-specific antibodies. (**C**) Silencing of HT-29/FLAGMusashi-1. HT-29/FLAGMusashi-1 cells were transfected with Musashi-1-specific siRNAs using LipoMax (Invitrogen) for 72 h. Total cell lysates were collected and subjected to immunoblotting analyses using a monoclonal anti-FLAG antibody. Musashi-1 knockdown in HT-29/FLAGMusashi-1 cells (upper left panel). HT-29/FLAGMusashi-1 cells were transfected with pre-designed siRNAs against Musashi-1 by liposome-mediated nucleic acid delivery. Relative fold change is indicated in the bar chart (lower left panel). Error bars indicate the mean ± SD from three independent experiments. ^#^p < 0.05. After 72 h, HT-29/FLAG and HT-29/FLAGMusashi-1 cells were subjected to CD44 expression analysis by flow cytometry (middle panels). Relative fold change is indicated in the bar chart (right panel). Error bars indicate the mean ± SD from three independent experiments. ^#^p < 0.05.

To determine whether the CRC stemness gene *Musashi-1* modulated CRC stemness properties, we established a series of Musashi-1 domain swap constructs that were sequenced and validated. We transfected 293 T cells with these constructs, and the expression patterns were validated by immunoblotting. HT-29, HCT-116, and LoVo cells were transfected with the FLAGMusashi-1 expression vector and selected by G418. FLAGMusashi-1 cells were validated by immunoblotting with anti-FLAG antibodies (Fig. 1A, left panel).

Because *Musashi-1* is a neuronal and colorectal epithelial cell stemness gene^35^, we examined spheroid formation to ascertain whether overexpression of Musashi-1 in HT-29, HCT-116, and LoVo cells could trigger CRC stemness properties. Indeed, spheroid formation was significantly increased in FLAGMusashi-1-overexpressing cells, including the HT-29, HCT-116, and LoVo cell lines, compared with that of their respective control cells (Fig. 1A, right panel).

These results indicated that Musashi-1 was associated with increased CSC properties. CD133, CD44, CD44v6, and Lgr5 were identified as CSC surface markers for CRCs, and CD44v6^+^ lineage-CSCs are associated with CRC malignancies and metastatic transformations. Therefore, we investigated whether overexpression of Musashi-1 promoted cancer cell stemness through lineage-dependent cellular transformations. Overexpression of FLAGMusashi-1 increased CD44, and the increase in CD44v6 expression was marginal (Fig. 1B).

To further determine whether Musashi-1 was sufficient for triggering CRC CD44^+^ populations, we knocked down Musashi-1 by delivering two specific small interferring RNAs to HT-29/FLAGMusashi-1 cells (Fig. 1C, left panel). The FLAGMusashi-1-overexpressing and knockdown HT-29 cells were subjected to CD44 expression analysis by flow cytometry. Musashi-1 knockdown reduced the number of CD44^+^ CRC cells, while overexpression of Musashi-1 increased these cells (Fig. 1C, right panel). These results indicated that Musashi-1 promoted the CD44^+^ CRC cell lineage and that these CRC cells may be highly metastatic.

**Figure 2:**
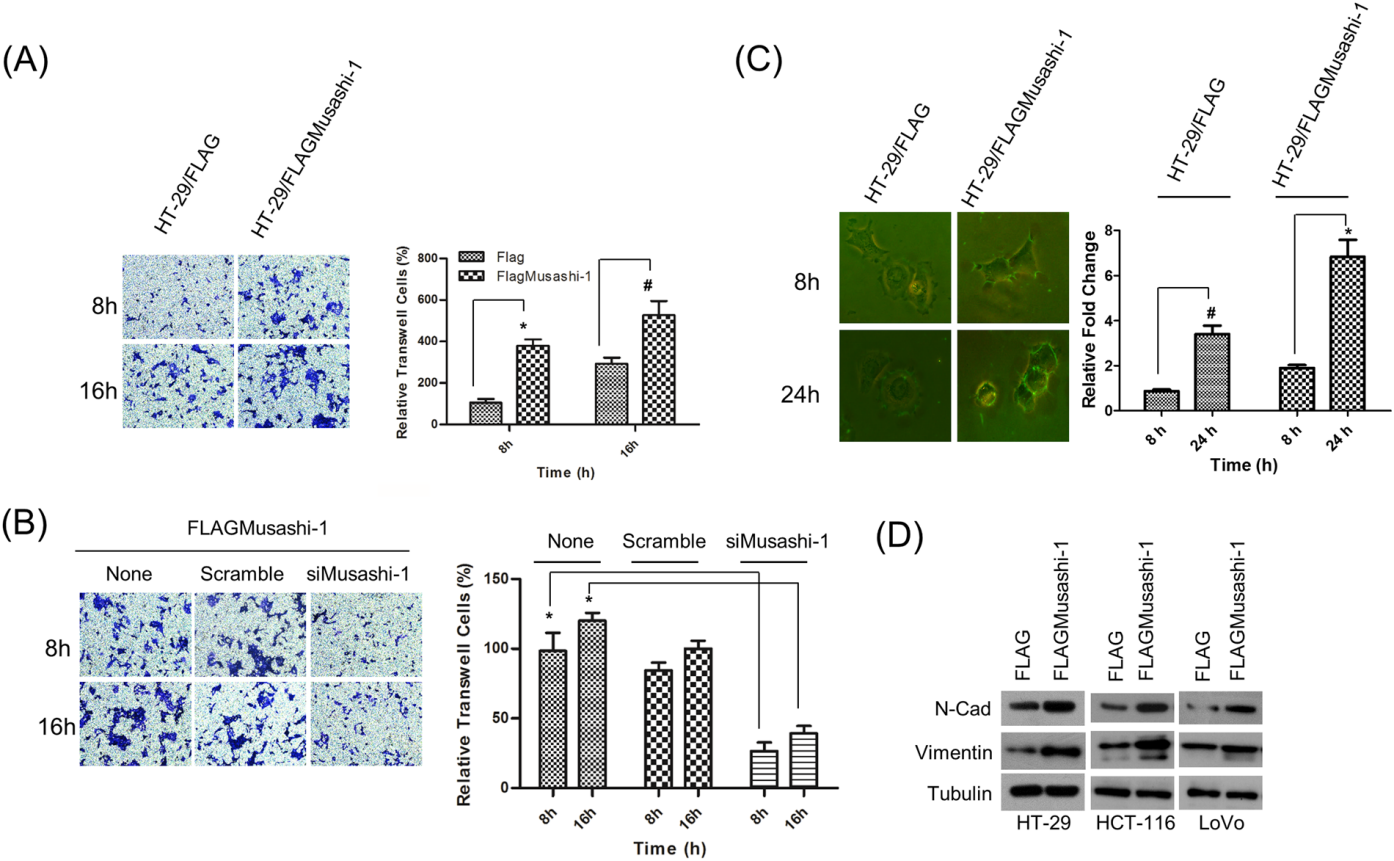
Musashi-1 promotes cell migration. (**A**) Increased migration in Musashi-1-overexpressing HT-29 cells. HT-29/FLAG and HT-29/FLAGMusashi-1 stably expressing HT-29 cells were subjected to the migration assay in 8 μm transwell chambers. Cells were fixed and stained by crystal violet after 8 and 16 h incubations (left panel). Relative migration was quantified, and the results are shown as a bar chart. Error bars indicate the mean ± SD from three independent experiments. *p < 0.01, ^#^p < 0.05 (right panel). (**B**) Cell migration is Musashi-1-dependent. HT-29/FLAGMusashi-1 cells were pretreated with Musashi-1-specific or scrambled siRNAs and then subjected to the transwell assay. Cells in the transwells were fixed and stained at the indicated time points (left panel). The results of the cell migration assay were quantified. Error bars indicate the mean ± SD from three independent experiments. *p < 0.01 (right panel). (**C**) Degradation of extracellular material by HT-29 cells overexpressing Musashi-1. HT-29/FLAGMusashi-1 and control HT-29/FLAG cells were plated on FITC-gelatin-coated coverslips. Cells were fixed at the indicated time points and images acquired using Olympus cellSens software v1.12 (left panel). Images were analysed for clear zone areas by ImageJ software. Clear zone areas were measured, normalized, and are presented as a bar chart. Error bars indicate the mean ± SD from three independent experiments. *p < 0.01, ^#^p < 0.05 (right panel). (**D**) Increased expression of metastasis-associated genes. Total cell lysates of HT-29, HCT-116, and LoVo cells/FLAG and HT-29, HCT-116, and LoVo cells/FLAGMusashi-1 cells were collected in RIPA buffer with protease and phosphatase inhibitors. Expression of N-cadherin (N-Cad) and vimentin was analysed by immunoblotting with specific antibodies.

We also assessed whether Musashi-1-overexpressing CRCs showed similar properties to those of CD44^+^ or CD44v6^+^ colorectal CSCs. The metastatic abilities of HT-29/FLAGMusashi-1 and HT-29/FLAG cells were determined by transwell assays measuring migration. At 16 h, there was a time-dependent increase in the migration of HT-29/FLAGMusashi-1 compared with that of HT-29/FLAG cells (2-fold). Additionally, the increase in the migration of HT-29/FLAGMusashi-1 cells at 8 h showed a onefold increase (Fig. 2A).

To further confirm that Musashi-1 was critical for HT-29 cell migration, we knocked down Musashi-1 by a siRNA specifically targeting the Musashi-1 mRNA coding region. The knockdown efficiency (approximately 80%) was determined by detecting FLAGMusashi-1 expression. Transwell assay results indicated that Musashi-1 enhanced HT-29 cell migration, and knockdown of Musashi-1 significantly attenuated cell migration (Fig. 2B). These results indicated that Musashi-1 promoted HT-29 metastasis.

Because Musashi-1 promoted cell migration in a time-dependent manner, we next determined whether it triggered the release of metastatic factors during cell migration. FITC-gelatin degradation experiments showed a significant increase in the clear zone area of HT-29/FLAGMusashi-1 compared with that of HT-29/FLAG cells (Fig. 2C). Furthermore, Musashi-1 promoted N-cadherin and vimentin expression levels in three CRC cell lines (Fig. 2D). These results indicated that Musashi-1 promoted CRC metastasis by enhancing extracellular matrix degradation activities.

**Figure 3:**
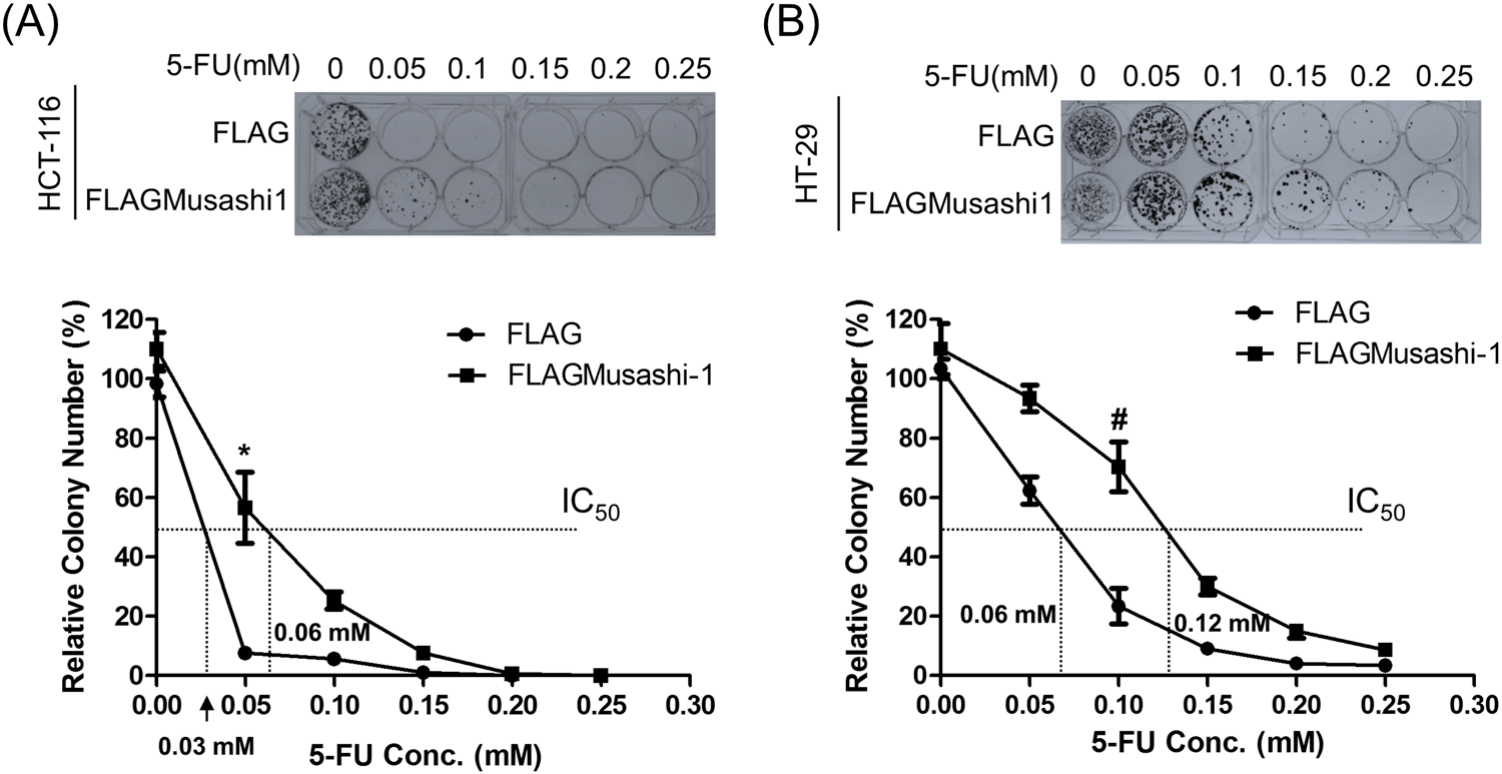
Overexpression of Musashi-1 enhances drug resistance in CRC cells. (**A**) FLAG- or FLAGMusashi-1-overexpressing HCT-116 were pretreated with increasing concentrations of 5-FU. Cells were then subcultured and seeded into new 6-well culture plates, fixed, and stained. Images were acquired using a Canon digital camera (upper panel). The relative cell survival curves were calculated and normalized. Square and circle indicate the mean ± SD from three independent experiments. *p < 0.01 (lower panel). (**B**) FLAG- or FLAGMusashi-1-overexpressing HT-29 cells were pretreated with increasing concentrations of 5-FU. Cells were then subcultured and seeded into new 6-well culture plates, fixed, and stained. Images were acquired using a Canon digital camera (upper panel). The relative cell survival curves were calculated and normalized. Square and circle indicate the mean ± SD from three independent experiments. ^#^p < 0.05 (lower panel).

Drug resistance and drug-induced cancer cell transformations remain the major clinical challenges in cancer therapies. Drug resistance in cancer cells is often associated with acquisition of cancer stem-like properties. To determine whether Musashi-1 mediated the drug resistance properties of CRCs, we treated Musashi-1-overexpressing HT-29 and HCT-116 (HCT-116/FLAGMusashi-1) cells with 5-fluorouracil (5-FU). The drug resistance properties of these cells were determined by a clonogenic assay. The results showed increased drug resistance (2-fold increase in the IC_50_) of both HT-29 and HCT-116 cells overexpressing Musashi-1 compared with that of their respective control cells (Fig. 3).

*Musashi-1* is a stemness gene of neuronal and colorectal epithelium cells; it is also an RNA-binding protein and localized predominantly in the cytosolic region. SGs enhance cellular survival by activating anti-apoptotic signalling pathways^36^. Hence, to determine whether Musashi-1 promoted CRC drug resistance by the formation of SGs, we exposed HT-116/FLAGMusashi-1 cells to arsenite, which increases the production of reactive oxygen species. The results showed that Musashi-1 cells formed granule-like intracellular structures after exposure to arsenite.

**Figure 4:**
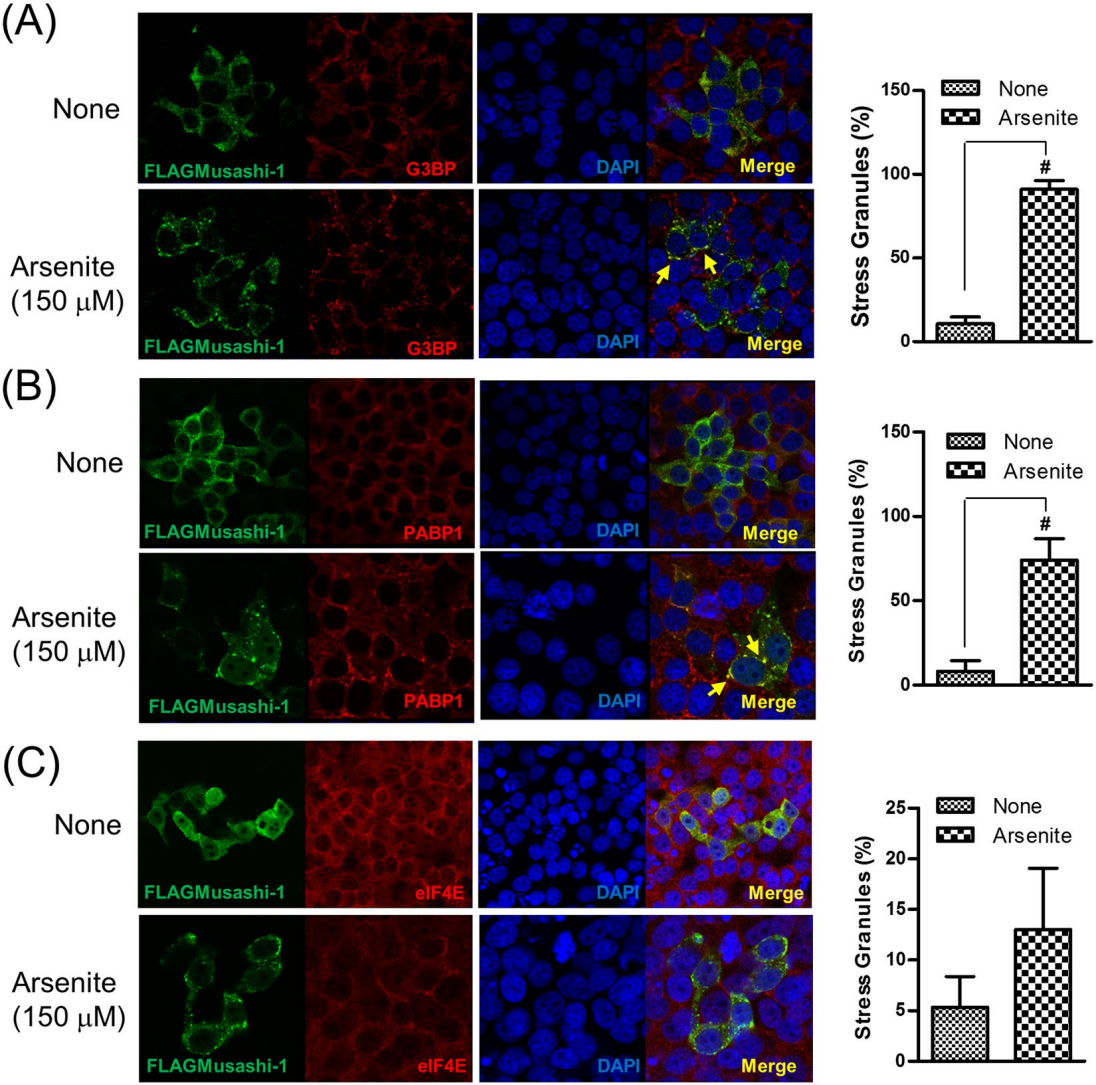
Musashi-1 forms SGs following treatment with arsenite. FLAGMusashi-1 transfected HT-29 cells were plated on coverslips and treated with 150 μM arsenite for 30 min. Cells were then fixed in 4% paraformaldehyde for 15 min at room temperature. After permeabilisation with 0.1% Triton X-100/PBS, FLAGMusashi-1-, PABP-, G3BP-, and eIF4E-specific antibodies were added to hybridization buffer at 4 °C overnight. The signals were amplified by Alexa488- or Alexa555-conjugated secondary antibodies. Images were acquired with a multiphoton confocal microscope. (**A**) Left panel: Co-localization of Musashi-1 and G3BP. Right panel: Statistical results of percentages of SG formation. Error bars indicate the mean ± SD from three independent experiments. ^#^p < 0.05. (**B**) Left panel: Co-localization of Musashi-1 and PABP1. Right panel: Statistical results of percentages of SG formation. Error bars indicate the mean ± SD from three independent experiments. ^#^p < 0.05. (**C**) Left panel: Co-localization of Musashi-1 and eIF4E. Arrow indicates Musashi-1 granules. Right panel: Statistical results of percentages of SG formation. Error bars indicate the mean ± SD from three independent experiments.

To further determine whether Musashi-1 intracellular punctate structures were induced by oxidative stress, we co-stained Musashi-1-overexpressing cells treated with arsenite for the known SGs markers G3BP (Fig. 4A), PABP1 (Fig. 4B), and eIF4E (Fig. 4C). The results indicated that Musashi-1 promoted SG formation in response to oxidative stress. However, other stress-inducing factors, such as aberrant shifts in temperature and unfolded protein responses, can also lead to the formation of SGs.

**Figure 5:**
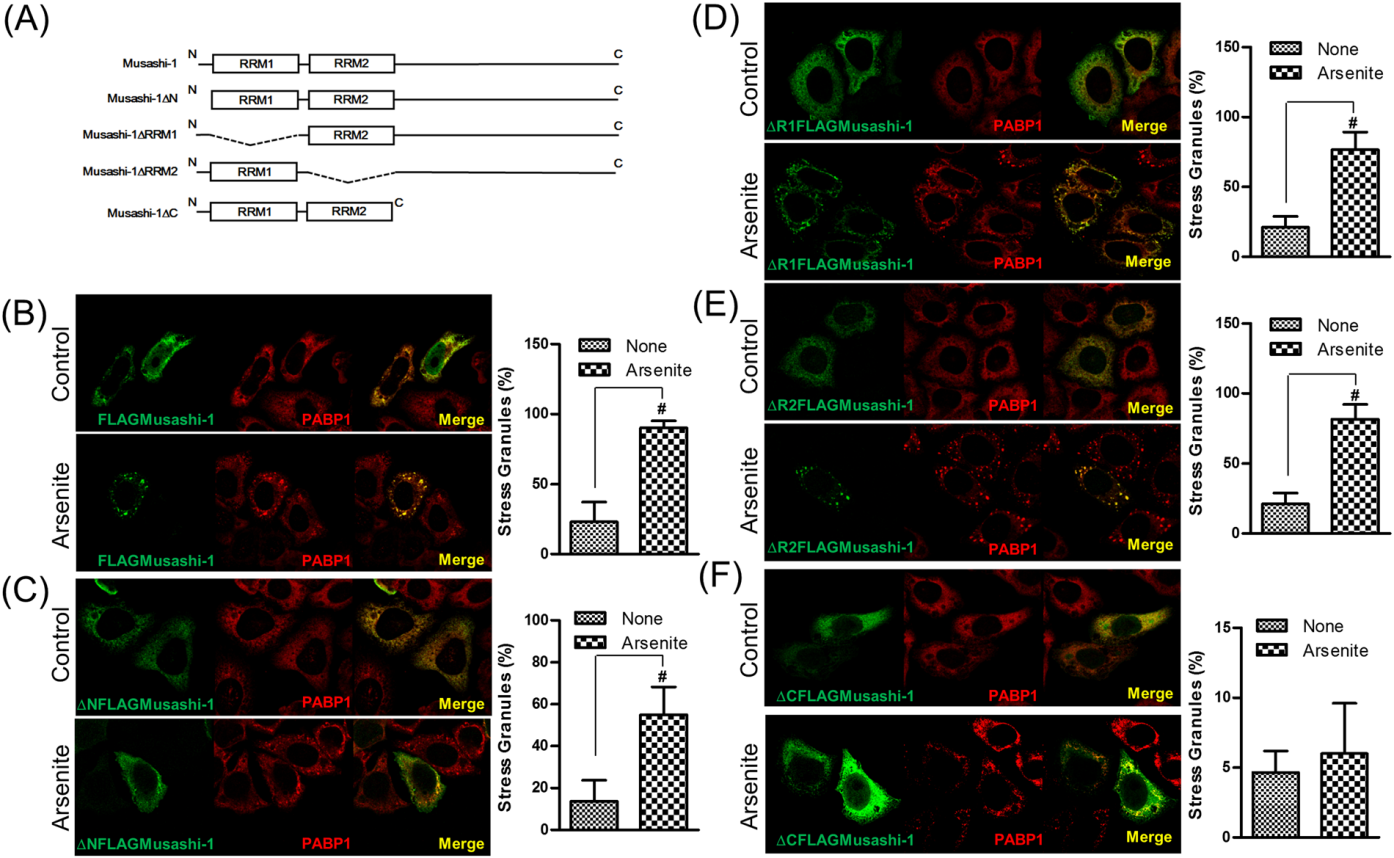
The Musashi-1 C-terminal domain is required for granule formation. HCT-116 cells were transfected with various Musashi-1 domain swap constructs. Forty-eight hours after transfections, cells were stimulated with arsenite (150 μM) for 30 min. Cells were then fixed in 4% paraformaldehyde at room temperature and subjected to immunostaining. (**A**) Schematic diagram of Musashi-1 domain swap constructs. (**B**) Left panel: Co-localization of FLAGMusashi-1 and PABP1. Right panel: Statistical results of percentages of SG formation. Error bars indicate the mean ± SD from three independent experiments. ^#^p < 0.05. (**C**) Left panel: Co-localization of ΔNFLAGMusashi-1 and PABP1. Right panel: Statistical results of percentages of SG formation. Error bars indicate the mean ± SD from three independent experiments. ^#^p < 0.05. (**D**) Left panel: Co-localization of ΔR1FLAGMusashi-1 and PABP1. (**E**) Left panel: Co-localization of ΔR2FLAGMusashi-1 and PABP1. Right panel: Statistical results of percentages of SG formation. Error bars indicate the mean ± SD from three independent experiments. ^#^p < 0.05. (**F**) Left panel: Co-localization of ΔCFLAGMusashi-1 and PABP1. Right panel: Statistical results of percentages of SG formation. Error bars indicate the mean ± SD from three independent experiments.

Musashi-1 contains several functional domains, including two RNA RRMs and a large C-terminal region. The previous experiments demonstrated that Musashi-1 formed SGs and that the RNP complex was critical for CRC acquisition of drug resistance. Serial domain swap constructs were generated to determine which functional domain of Musashi-1 was required for the formation of SGs. These constructs were introduced into HT-29 cells by liposome-mediated transfection. Musashi-1 granule formation was assessed after exposing cells to 150 μM arsenite for 30 min. Although RRMs were critical for the formation of the RNP complex due to their RNA-binding activities, our results showed that the C-terminal domain played a crucial role in Musashi-1 granule formation (Fig. 5). These results indicated that Musashi-1 formed SGs by protein-protein interactions via the C-terminal region and that the Musashi-1 C-terminal domain contributed to the formation of SGs.

**Figure 6:**
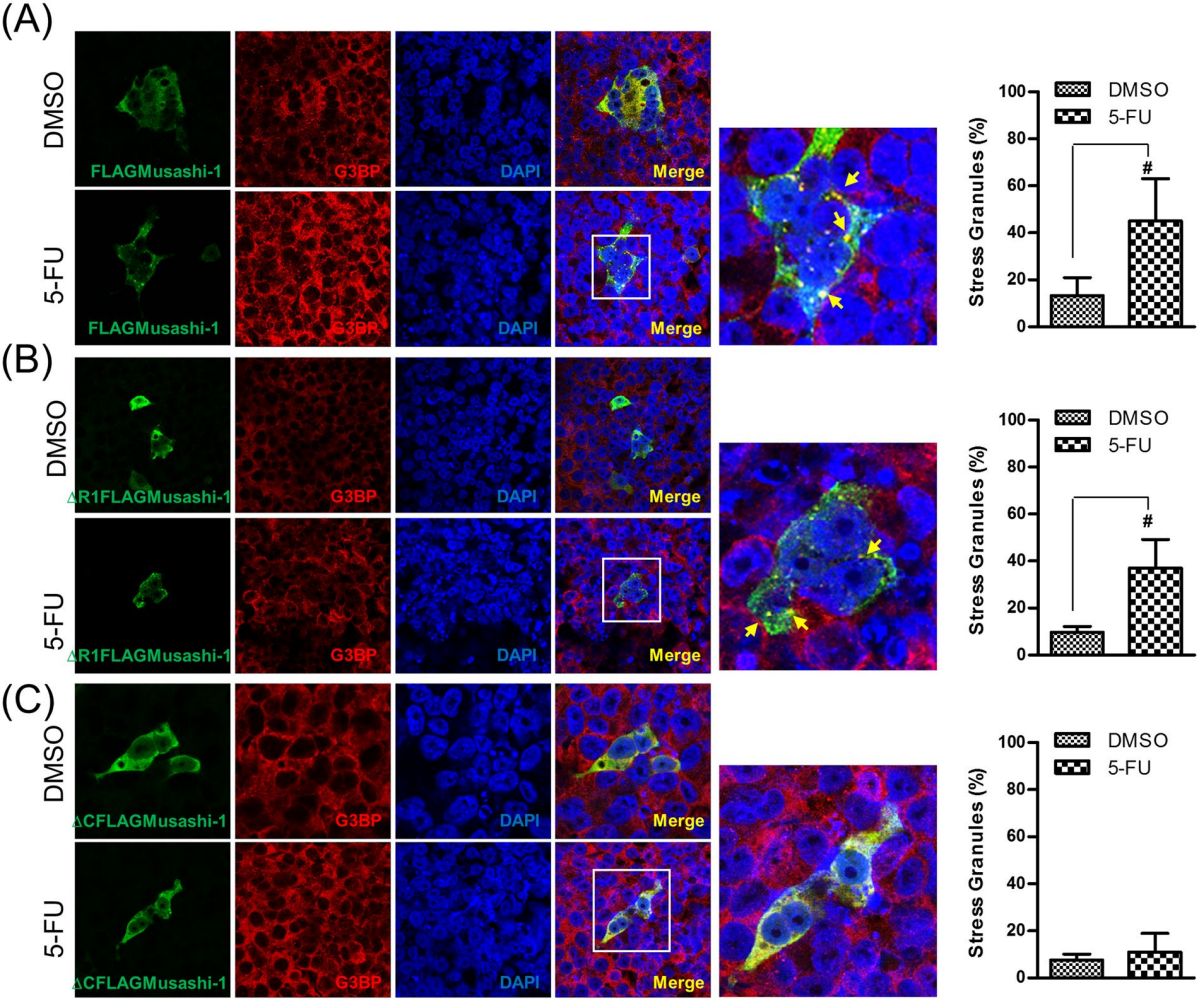
5-FU induces Musashi-1 granule formation. HCT-116 cells were transfected with FLAGMusashi-1 or ΔCFLAGMusashi-1 domain swap constructs. Forty-eight hours after transfections, cells were treated with 5-FU (400 μM) for 24 h. Cells were then fixed in 4% paraformaldehyde at room temperature and subjected to immunostaining as described in the Methods. (**A**) Left panel: Co-localization of FLAGMusashi-1 and G3BP. Right panel: Statistical results of percentages of SG formation. Error bars indicate the mean ± SD from three independent experiments. ^#^p < 0.05. (**B**) Left panel: Co-localization of ΔR1FLAGMusashi-1 and G3BP. Right panel: Statistical results of percentages of SG formation. Error bars indicate the mean ± SD from three independent experiments. ^#^p < 0.05. (**C**) Left panel: Co-localization of ΔCFLAGMusashi-1 and G3BP. Right panel: Statistical results of percentages of SG formation. Error bars indicate the mean ± SD from three independent experiments. Arrow indicates Musashi-1 granules.

To determine whether a clinical anticancer drug, 5-FU, could trigger Musashi-1 granule formation, we treated HCT-116 cells transfected with the FLAGMusashi-1 expression vector with this drug. Interestingly, 5-FU triggered the formation of Musashi-1 granules, and these granules co-localized with G3BP, a SG marker (Fig. 6A). To further determine which domain was critical for 5-FU-stimulated Musashi-1 granule formation, we examined whether this effect was also C-terminal-dependent. As expected, the Musashi-1 C-terminal was essential for 5-FU-stimulated Musashi-1 granule formation (Fig. 6B). The results indicated that 5-FU triggers the formation of Musashi-1-positive SGs in CRCs. These findings were consistent with previous reports showing that 5-FU promoted the formation of SGs^37^. Furthermore, the Musashi-1 C-terminal domain was required for the formation of 5-FU-induced SGs. Because SGs were reported to have anti-apoptotic activity, we next determined whether CRC drug resistance was involved in the anti-apoptotic effects of Musashi-1.

**Figure 7:**
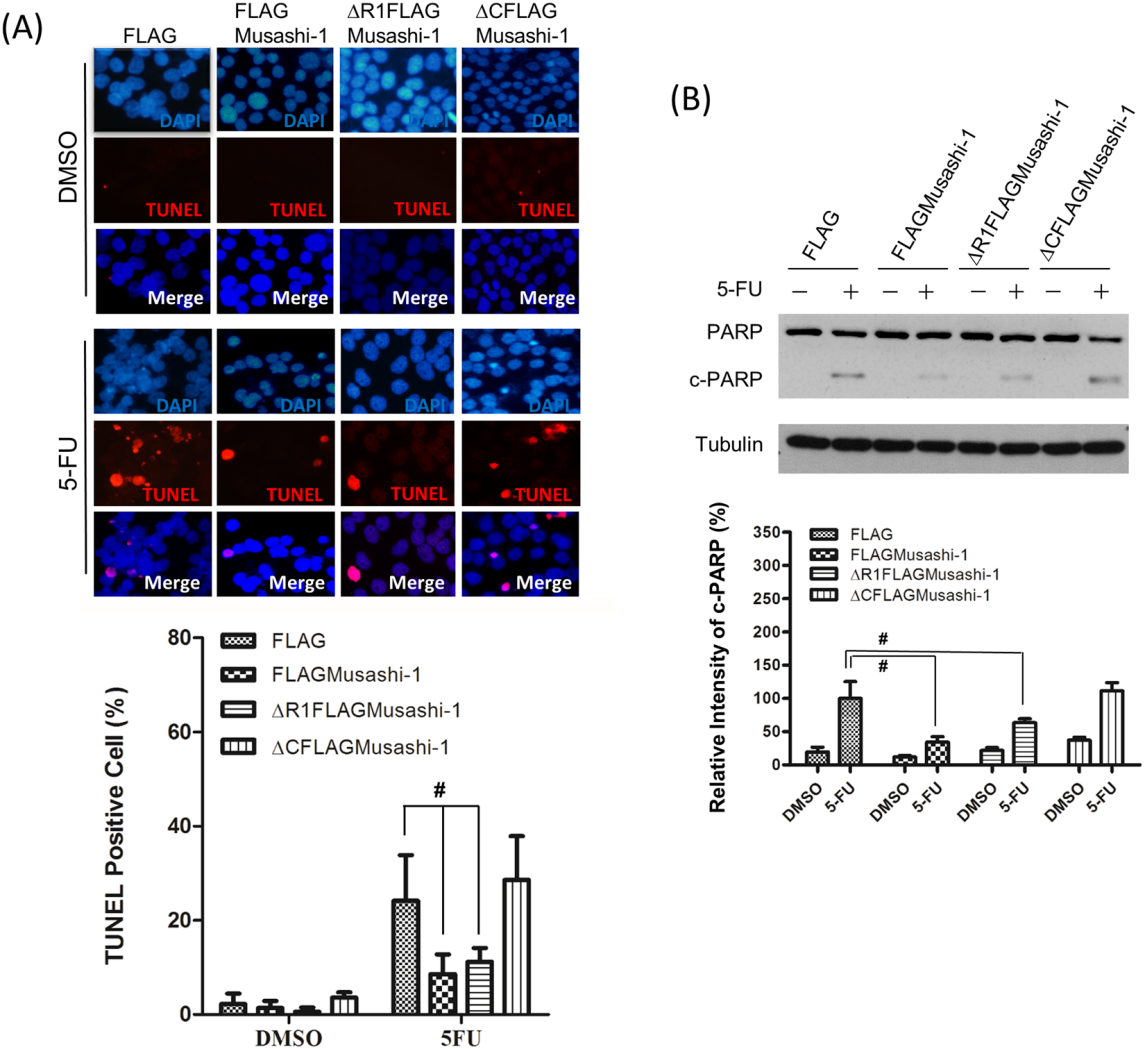
Musashi-1 inhibits 5-FU-induced apoptosis in HCT-116 cells. (**A**) Upper panel: FLAG, FLAGMusashi-1, ΔR1FLAGMusashi-1, and ΔCFLAGMusashi-1 HCT-116 stable clones were seeded on 22 mm × 22 mm coverslips and treated with 5-FU (400 μM) for 24 h. Cells were fixed in 4% paraformaldehyde and subjected to the TUNEL assay as described in the Methods. Lower panel: Statistical results of percentages of TUNEL positive cells. Error bars indicate the mean ± SD from three independent experiments. ^#^p < 0.05. (**B**) Upper panel: FLAG, FLAGMusashi-1, ΔR1FLAGMusashi-1, and ΔCFLAGMusashi-1 HCT-116 stable clones were treated with or without 5-FU (400 μM) for 24 h. Total cellular proteins were isolated and subjected to immunoblotting with antibodies specific to PARP. Increased cleaved PARP (c-PARP) signals were observed in ΔCFLAGMusashi-1 HCT-116 stable clones. Tubulin indicates Tubulin protein as an internal control. Lower panel: Statistical results of percentages of relative intensity of c-PARP. Error bars indicate the mean ± SD from three independent experiments. ^#^p < 0.05.

The terminal deoxynucleotidyl transferase dUTP nick end labelling (TUNEL) assay revealed that HT-116/FLAGMusashi-1 cells incubated with 5-FU showed less apoptosis than that of HCT-116 cells without FLAGMusashi-1 (Fig. 7A). To examine the function of the Musashi-1 C-terminal domain with regard to its anti-apoptotic properties in CRCs, we established stable FLAG, FLAG-Musashi-1, and C-terminal domain-truncated HCT-116 cells. The FLAG and Musashi-1 C-terminal domain-deleted stable clones showed an approximately 2-fold increase in CRC apoptosis induced by 5-FU treatments, while in Musashi-1-overexpressing cells, there was little increase in PARP cleavage (Fig. 7B). Together, these results demonstrated that Musashi-1 triggers CRC drug resistance by the formation of SGs. Furthermore, the C-terminal domain of Musashi-1 may have a critical role in mediating SG formation. The Musashi-1 C-terminal domain has an endogenous binding site for interaction with other RNA-binding proteins, such as Lin28^38^. Thus, Musashi-1 may be recruited to SGs by Lin28 or PABD^39^ domain in Musashi-1 may also regulates Musashi-1 containing SGs formations by direct binding of RNAs. The Musashi-1 C-terminal may interact with factors critical for drug resistance.

**Figure 8:**
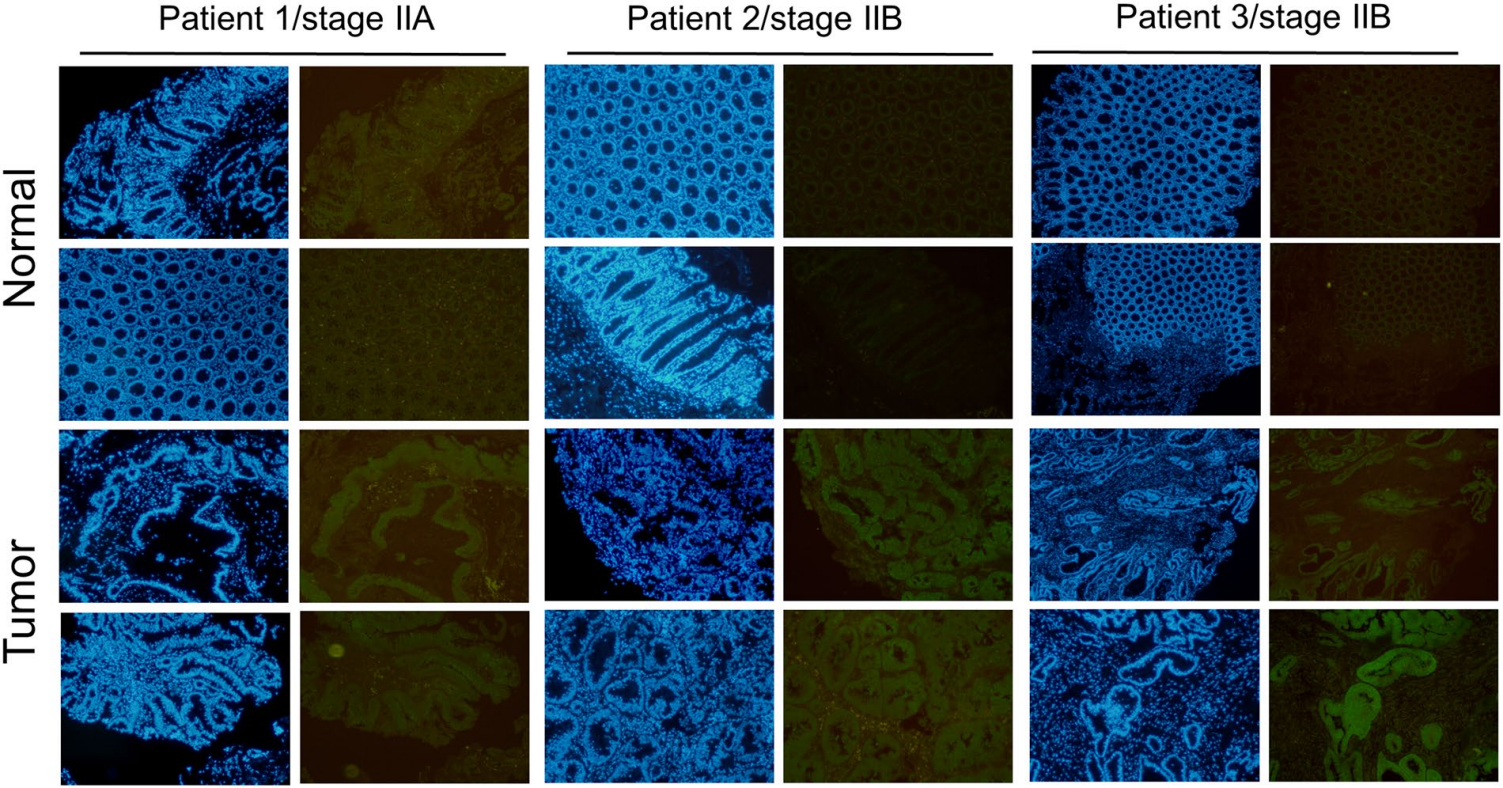
Increased Musashi-1 expression in CRC cells. Paired clinical samples (one stage IIA and two stage IIB samples) of normal and CRC tissues were subjected to Musashi-1 immunohistochemical staining as described in the Methods. Relative Musashi-1 expression levels were detected by a Musashi-1-specific antibody and fluorescence dye-conjugated secondary antibodies.

Stage IIA and Stage IIB are pre-metastasis stages of CRC; however, there are still clinical cases of Stage IIA and Stage IIB with malignant CRC transformations. Our previous data indicated that Musashi-1 has a critical role in modulating CRC malignancy, including CD44-positive CRC traits, such as transformation, drug resistance, and cell migration. Therefore, we examined whether Musashi-1 expression is increased in the tumour section of CRC clinical samples. The expression of Musashi-1 in clinical CRC samples was assessed by immunohistochemical staining. Musashi-1 expression levels were increased in the tumour but not normal colon tissues (Fig. 8). These results indicated a correlation between Musashi-1 expression and stage IIA and stage IIB CRC progression.

## Discussion

The clinical challenges of CRC, including drug resistance and metastasis, are major obstacles to effective treatments. CSCs are believed to contain niches of cancer cell populations that have stem cell properties. CSCs were discovered in CRCs and found to express specific surface markers. For example, CRCs with CD44, CD44v6, CD133, Lgr5, and Eph2 were reported to have CSC properties. These findings indicated that the colorectal CSC populations are highly heterogeneous and that different CSCs might have different properties. For example, CD44^+^ CRCs are enriched in 5-FU-damaged intestinal tissue. Eph2 expression predicts CRC relapse, and CD44v6^+^ CRCs are highly associated with metastasis. However, studies on intracellular factors regulating colorectal CSCs are lacking.

Recently, in addition to β-catenin, Musashi-1 has been reported as a critical oncoprotein for CRC^40^. Thus, we investigated how Musashi-1 regulates colorectal CSC properties and malignant transformation. Musashi-1 is an established downstream regulator of the Notch and Numb signalling pathways. The Notch pathway is essential for maintaining colorectal CSC niches. Here, we report SG formation as a novel function of Musashi-1 in colorectal cancer cells—or in intestinal tissue—and that this intracellular RNP complex contributes to drug resistance and metastasis.

The heterogeneity of tumour cells may allow for selection of drug-resistant CRCs from the surviving population. 5-FU is a first line drug for CRCs and blocks DNA synthesis by inhibiting thymidine kinase. Multiple mutations are present in drug-resistant CRCs. Our results showed that among the colorectal CSC surface markers we examined, CD44, CD133, Lgr5, and overexpression of Musashi-1 selectively promoted CD44^+^ colorectal CSC traits.

There are multiple CSC lineages, and we postulated that Musashi-1 was linked to the CD44^+^ colorectal CSC traits. Knockdown of Musashi-1 attenuated CD44^+^ CRC populations. Interestingly, staining of intestinal ducts damaged by high doses of 5-FU showed increased levels of Musashi-1 and CD44^+^ cells. These results indicated that Musashi-1 and CD44^+^ could be linked to CRC 5-FU resistance and suggested that 5-FU triggered SG formation^37^.

Musashi-1 is a RNA-binding protein located intracellularly in the cytosol. Increasing evidence suggests that SGs have anti-apoptotic activities by inhibiting the Rack1 or p38 signalling pathways^41^. Inhibition of the mTORC1 pathway also prevents apoptosis in cancer cells^42^. However, inhibition of TIA-1, an SG marker, abolishes SG formation and enhances apoptosis^43^. Musashi-1 and Musashi-2 are functionally redundant, with similar transcripts, and promote tumourigenesis independent of β-catenin. Interestingly, in a Musashi-1/Musashi-2 double knockout animal model, the AOM-DSS colitis protocol failed to induce CRC. These findings indicate that Musashi family oncoproteins may be linked to inflammation and CRC. Musashi-1 or Musashi-2 is likely one of the regulatory components of the inflammasome, while the inflammasome and SGs may be associated following stress stimulation, such as inflammation-induced increases in intracellular reactive oxygen species. Furthermore, the C-terminal domains of Musashi-1 and Musashi-2 are different. Therefore, elucidating the interactions of Musashi-1 or possibly Musashi-2 SG formation and environmentally induced stress would be interesting.

Musashi-1 contains two RRMs and functions as a regulatory factor that links extracellular microenvironments to intracellular signalling pathways through Notch signalling pathways. In addition, Musashi-1 has a protective function in spermatogenesis against heat stress through the formation of SGs^44^. Furthermore, Musashi-1 inhibits signalling networks by directly binding to Numb mRNA via the RRMs. Intracellular targeting creates cellular toxicities, and nonspecific intracellular stress responses are triggered by drug treatments. Indeed, Musashi-1 promotes glioblastoma (GBM) drug resistance^45^. How these stress responses contribute to cancer cell plasticity and the acquisition of drug resistance is an unanswered question.

Whether Musashi-1 exerts its cancer-promoting activities through SGs is unclear. Although RRM domains are essential for Musashi-1-mediated SG formation, our results show that the C-terminal domain is required for this process as well as for HCT-116 cell migration. The C-terminal domain interacts with multiple regulatory proteins, including Lin28 and PABP^38^. Lin28 is a critical oncogene that regulates cancer activities through let-7 microRNA biogenesis, and PABP is a SG marker. Lack of PABP would also reduce the granule formation of Musashi-1 since Musashi-1 would not be recruited to SGs by interacting with PABP. Besides, PolyA-binding domain (PABD) is located in the C-terminal domain of Musashi-1 and PABD domain may regulate SGs formation^39^. Furthermore, the colocalization of Musashi-1 and eIF4E is weaker than the Musashi-1 association with G3BP or PABP. This is probably due to Musashi-1 C-terminal domain interactions with Lin28 or PABP for SG formations rather than eIF4E. Besides RRMs, PABD domain may have critical role in RNA binding in stressed condition. Thus, there may be alternative mechanisms for Musashi-1 recruitment to SGs in response to different environmental stresses. Different SGs may also have different functions in regulating CRC cell properties. CRC patient samples stained for Musashi-1 revealed increased expression compared to that of normal colorectal tissues. These findings are consistent with the association of Musashi-1 with CRC progression. Overall, we showed that increased Musashi-1 expression could trigger CRCs to acquire drug resistance and metastatic properties and thereby contribute to clinical treatment difficulties. This suggests that Musashi-1 is a potential CRC therapeutic target.

## Methods

### Plasmid constructions and small interfering RNA knockdown

*Musashi-1* (NM_002442.3) was amplified with M1 forward and M1 reverse primers by Q5 high fidelity DNA polymerase (NEB, Ipswich, MA) from cDNA library prepared from HT-29 cells total RNAs with Superscript III (Thermo Fisher Scientific Waltham, MA). For subcloning full length FLAGMusashi-1 into Hind III and Xba I sites of p3xFLAG-CMV-10 (E7658, Sigma, St. Louis, MO). Full length FLAGMusashi-1 was amplified with Q5 high fidelity DNA polymerase (NEB, Ipswich, MA) with primers: Musashi-1 forward and Musashi-1 reverse. The amplified full length Musashi-1 fragments with HindIII/Xba I sites were then subcloned into Hind III/Xba I digested p3xFLAG-CMV-10 by HiFi DNA Assembly Master Mix (NEB, Ipswich, MA). For ΔCFLAGMusashi-1 constructions, full length Musashi-1 was amplified with primers: Hind III/ΔCMusashi-1 forward and Xba I/ΔCMusashi-1 reverse. The amplified ΔCFLAGMusashi-1 was subcloned into Hind III/Xba I site of p3xFLAG-CMV-10. For ΔNFLAGMusashi-1 constructions, full length Musashi-1 was amplified with primers: Hind III/ΔNMusashi-1 forward and Xba I/Musashi-1 reverse. The amplified ΔNFLAGMusashi-1 was subcloned into Hind III/Xba I site of p3xFLAG-CMV-10. For ΔR1FLAGMusashi-1 constructions, p3xFLAG-CMV-10 vector with full length FLAGMusashi-1 was amplified with primers: ΔR1 reverse and ΔR1 forward with Q5 high fidelity DNA polymerase (NEB, Ipswich, MA). Amplified fragments with 15 bps of overlapping sequences were recovered and ligated by HiFi DNA Assembly Master Mix (NEB, Ipswich, MA). For ΔR2FLAGMusashi-1 constructions, p3xFLAG-CMV-10 vector with full length FLAGMusashi-1 was amplified with primers: ΔR2 reverse and ΔR2 forward with Q5 high fidelity DNA polymerase (NEB, Ipswich, MA). Amplified fragments with 15 bps of overlapping sequences were recovered and ligated by HiFi DNA Assembly Master Mix (NEB, Ipswich, MA). All constructed clones were validated by sequencing and the expression of encoded protein were examined and validated by immunoblotting. Summaries of primer lists and sequences are shown below:

M1 forward:

5′-ATGGAGACTGACGCGCCCCAGCCCG-3′

M1 reverse:

5′-TCAGTGGTACCCATTGGTGAAGGCT-3′

Musashi-1 forward:

5′-TGACGATGACAAGCTATGGAGACTGACGCGCCCCAGCCCG-3′

Musashi-1 reverse:

5′-CCCCGGATCCTCTAGTCAGTGGTACCCATTGGTGAAGGCT-3′

Hind III/ΔCMusashi-1 forward:

5′-TGACGATGACAAGCTATGGAGACTGACGCGCCCCAGCCCG-3′

Xba I/ΔCMusashi-1 reverse:

5′-CCCCGGATCCTCTAGTTTCTTACATTCCACCATTTTGTTG-3′

Hind III/ΔNMusashi-1 forward:

5′-TGACGATGACAAGCTATGTTCATCGGGGGACTCAGTTGGC-3′

Xba I/Musashi-1 reverse:

5′-CCCCGGATCCTCTAGTCAGTGGTACCCATTGGTGAAGGCT-3′

ΔR1 reverse:

5′-CTTGCAGGGGTCGTGCGGCGAGTCC-3′

ΔR1 forward:

5′-CACGACCCCTGCAAGCCTCGGCGAGCACAGCCCAAGATGG-3′

ΔR2 reverse:

5′-CTTCTTCGTTCGAGTCACCATCTTG-3′

ΔR2 forward:

5′-ACTCGAACGAAGAAGGCTCAGCCAAAGGAGGTGATGTCGC-3′

For Musashi-1 knockdown, Musashi-1 was knockdown by two specific siRNAs:

siRNAi#1: sense 5′-CAUGCUGAUGUUUGACAAA[dT][dT]-3′/anti-sense 5′-UUUGUCAAACAUCAGCAUG[dT][dT]

siRNA#2: sense 5′-GGUUCGGGUUUGUCACGUU[dT][dT]-3′/anti-sense 5′-AACGUGACAAACCCGAACC[dC][dT]).

Transfection of siRNAs including scramble siRNA and Musashi-1 specific siRNAs were performed with RNAiMax reagent (Thermo Fisher Scientific Waltham, MA) for 72 h.

### Cell Culture, Stable Clone Selection, and Clinical Sample Collection

CRC cells were cultured and maintained in McCoy’s 5a medium (Thermo Fisher Scientific Waltham, MA) supplemented with 10% foetal bovine serum (Thermo Fisher Scientific Waltham, MA) and penicillin/streptomycin (Thermo Fisher Scientific Waltham, MA). For the selection of stable clones, 1 × 10^5^ HT-29, HCT-116 or LoVo cells were transfected with control (FLAG) or Musashi-1-expressing (FLAGMusashi-1) plasmids by liposome-mediated DNA delivery using Lipofectamine 2000 (Thermo Fisher Scientific Waltham, MA). Forty-eight hours after transfection, the culture medium was replaced with medium containing 5 mg/mL G418 (Sigma, St. Louis, MO) for 4 weeks. Clones were isolated, and the expression of FLAG or FLAGMusashi-1 was determined by immunoblotting with an anti-FLAG antibody (Sigma, St. Louis, MO). Clinical CRC sample collection was approved and supervised by the Institutional Review Board (IRB) of Chung Shang Medical University Hospital. All methods were performed in accordance with the relevant guidelines and regulations. Informed consent was obtained from all subjects.

### Spheroid Formation and Transwell Assay

Cells (1 × 10^3^) were cultured for 2 weeks in spheroid formation medium supplemented with B27 (Thermo Fisher Scientific Waltham, MA) and N2 (Thermo Fisher Scientific Waltham, MA). For the transwell assay, HT-29 cells were seeded in transwells at a density of 1 × 10^5^ cells/well for 16 h. Cells were then fixed and stained. Cell numbers were quantitated by ImageJ software.

### Immunoblotting and Immunofluorescence Staining

Cells were lysed with RIPA buffer (50 mM Tris-HCl, pH 7.4, 1% NP-40, 0.5% sodium deoxycholate, 0.1% SDS, 150 mM sodium chloride, 2 mM EDTA). Total protein concentrations were determined by the Bradford method at a wavelength of 595 nm. Immunoblots were performed with specific antibodies using total protein immobilized on polyvinylidene fluoride membranes. In brief, prior to incubation with primary antibodies, polyvinylidene fluoride membranes were preincubated with 10% skim milk for 1 h and then with the specific antibodies described in the Results. After the samples were washed, secondary antibodies were added to the incubation buffer. Immunoblotting signals were detected by enhanced chemiluminescence (Thermo Fisher Scientific Waltham, MA). The anti-FLAG antibodies (F3165 Monoclonal ANTI-FLAG® M2 antibody and F7425 - ANTI-FLAG® antibody) were purchased from Sigma-Aldrich, Taiwan. Anti-Musashi-1 [Musashi-1 (D46A8) Rabbit mAb #5663] for clinical sample staining was purchased from Cell Signaling Technology (Danvers, MA 01923). Antibodies against PABP1 (#4992), N-Cadherin (D4R1H, #13116), Vimentin (D21H3 #5741), CD133 (A3G6K, #5860), CD44 (156-3C11, #3570), and eIF4E (C46H6, Rabbit mAb #2067) were purchased from Cell Signaling Technology. Lgr5 (Clone #707042), CD133 (Clone # 170411), and CD44v6 antibodies (Clone #2F10) were purchased from R&D Systems, Minneapolis, MN 55413.

For immunofluorescence staining, cells were cultured on acid-pretreated and sterilized coverslips in 6-well culture plates. Cells were fixed in 4% paraformaldehyde for 15 min at room temperature. After fixation, cells were permeabilised with 0.1% Triton X-100 in phosphate-buffered saline (PBS) for 10 min and then incubated with 1% BSA (Sigma, St. Louis, MO) at room temperature for 1 h. Primary antibodies (1:500) were incubated with fixed and permeabilised cells overnight at 4 °C. The cells were then washed three times with 0.1% Triton X-100 in PBS. Fluorescence probe-conjugated secondary antibodies (1:500) were incubated with cells for 1 h at room temperature. The coverslips were air-dried, preserved with anti-fade mounting solution, and covered with glass slides. Images were acquired by a Leica TCS-SP5-X AOBS confocal microscope system (Leica, Wetzlar and Mannheim, Germany).

### FITC-gelatin Degradation Assay

FITC-gelatin (Thermo Fisher Scientific Waltham, MA) was used to coat acid-pretreated 22 mm × 22 mm coverslips. Cells (1 × 10^3^) were seeded on the FITC-gelatin-coated coverslips. The clear zone of FITC-gelatin was determined using an Olympus DP-80 microscope image system (Olympus, Tokyo, Japan). Images were quantitated by ImageJ software.

### Clonogenic Assay

CRC cells from FLAG and FLAG-Musashi-1 stable clones were pretreated with different concentrations of 5-FU (R&D Systems, McKinley Place NE, MN) for 48 h. Cells were then detached from the culture dishes by 0.25% trypsin. The detached cells were seeded into 6-well plates at 1000 cells per well. After 2 weeks of culture, cell colonies were fixed, stained with crystal violet, and counted.

### Flow Cytometry Assay

Cells were detached from culture dishes with 0.05% trypsin at 37 °C for 5 min and were then incubated for 10 min in medium containing 10% foetal bovine serum. After centrifugation, the cells were incubated with an FITC-conjugated anti-CD44 antibody (clone MEM-85, SAB4700182, Sigma, St. Louis, MO) for 2 h at 4 °C. Flow cytometry analysis of the cells was performed using a ACEA NovoCyte flow cytometer (ACEA Bioscience, San Diego, CA).

### TUNEL Staining

HCT-116 stable cells (FLAG, FLAGMusashi-1, ΔR1FLAGMusashi-1 and ΔCFLAGMusashi-1) were plated on coverslips. Cells were then stimulated with or without 5-FU (400 μM) for 24 h as indicated. Cells were fixed in 4% paraformaldehyde for 15 min at room temperature. After permeabilisation three times with 0.1% Triton X-100/PBS for 10 min and blocking, the cells were incubated with terminal deoxynucleotidyl transferase (NEB, Ipswich, MA) and digoxigenin-11-dUTP (Roche Life Science, Taipei, Taiwan) at 37 °C for 1 h. The cells were then incubated with anti-FLAG- and anti-digoxigenin-specific IgG. Alexa-488 (Thermo Fisher Scientific Waltham, MA) or Alexa-545 (Thermo Fisher Scientific Waltham, MA) fluorescence dye-conjugated anti-mouse or anti-rabbit IgG was incubated with fixed cells. Images were acquired by an Olympus DP-80 microscope image system. (Olympus, Tokyo, Japan). Images were processed and merged by ImageJ software.
